# CCR2 overexpressing gingiva mesenchymal stem cells provide high intestinal regeneration in a rat model of ulcerative colitis

**DOI:** 10.1371/journal.pone.0325566

**Published:** 2025-06-05

**Authors:** Leyla Tekin, Deniz Genç

**Affiliations:** 1 Department of Pathology, Faculty of Medicine, Muğla Sıtkı Koçman University, Muğla, Turkey; 2 Faculty of Health Sciences, Muğla Sıtkı Koçman University, Muğla, Turkey; 3 Research Laboratories Center, Immunology and Stem Cells Laboratory, Muğla Sıtkı Koçman University, Muğla, Turkey; King Abdulaziz University Faculty of Medicine, SAUDI ARABIA

## Abstract

**Background/aims:**

Ulcerative colitis (UC) is a chronic inflammatory disease affecting the colon. Mesenchymal stem cells (MSCs) are candidates for use in inflammatory diseases with their tissue regeneration and anti-inflammatory capabilities. Chemokine receptor overexpression on MSCs is an effective strategy for the migration of MSCs into inflammatory tissue in high amounts. In this study, the anti-inflammatory and regenerative effects of genetically CCR2 overexpressed gingiva MSCs (GMSCs) on the inflamed colon in UC were studied.

**Materials and methods:**

GMSCs were transduced with a lentiviral vector for CCR2 overexpression. The UC experimental model was induced with a single intrarectal administration of 4% w/w acetic acid. Colon tissues were analyzed for IFN or IL-17 secreting CD4+  T lymphocytes via flow cytometry and lymphocytic infiltration, fibrosis, and ulcers by histopathologic evaluation. Homing analysis of GMSCs was done by analyzing fluorescence intensity under the fluorescence microscope.

**Results:**

GMSCs and CCR2^+^GMSCs equally downregulated colonic IFN-γ or IL-17 secreting CD4+ T lymphocytes ratios compared to the untreated group (p < 0.05). Fluorescence intensity of labeled cells was significantly high in colon tissues of CCR2^+^GMSCs administered rats (61.2 ± 13.7%) compared to GMSCs administered rats (19.6 ± 9.8%) (p < 0.05). In addition, mucosal integrity significantly increased and fibrosis and ulcers notably decreased in CCR2^+^GMSCs administered rats compared to GMSCs administered rats (p < 0.05).

**Conclusion:**

The overexpression of CCR2 on GMSCs increases migration to the inflammatory colon tissue, which has a high regenerative effect in UC. Overexpression of CCR2 on GMSCs may be an alternative to cellular therapies in UC.

## Introduction

Inflammatory bowel disease (IBD) is characterized by recurrent inflammation of the gastrointestinal tract caused by an abnormal immune response against the intestinal microflora [1]. Basically, IBD is of two types; Ulcerative colitis and Crohn’s disease. Ulcerative colitis is a chronic inflammatory disease affecting the colon and its incidence is increasing worldwide. Its pathogenesis includes genetic predisposition, epithelial barrier defects, dysregulated immune responses, and environmental factors [2]. Patients with ulcerative colitis have mucosal inflammation that begins in the rectum and can extend continuously to the proximal segments of the colon. In the course of the disease, there are acute attacks, life-threatening complications, and the risk of developing colorectal cancer [3]. For this reason, it is important to develop appropriate treatments that target inflammatory responses and tissue regeneration.

In several experimental studies conducted in recent years, it has been shown that systemic injection of mesenchymal stem cells (MSCs) isolated from bone marrow, umbilical cord, or adipose tissue gives effective results in IBD and suppresses intestinal inflammation [4]. Among all MSCs sources, dental MSCs are candidates for use in UC with their high regeneration and anti-inflammatory abilities as well as their easy and rapid isolation [5]. Dental tissues such as dental pulp, dental follicle, periodontal ligament, and gingiva are rich sources of MSCs and have high immunomodulatory capability on the activated lymphocytes and have high regenerative capacity compared to bone marrow or adipose tissue MSCs [6]. In a previous study, it was demonstrated that MSCs isolated from dental follicle tissues have a modulatory effect on peripheral mononuclear cells and inflammatory intestinal tissue mononuclear cells of Crohn’s patients [7]. As the previous study showed that dental follicle MSCs exhibited a strong anti-inflammatory response, in the present study we targeted to investigate immunomodulatory and regenerative effects of gingiva MSCs which is a rich source for dental MSCs and also can be isolated in high amounts. The main problem observed in the studies carried out to date is the migration of MSCs in insufficient amounts to the target tissue after intravenous or intraperitoneal injection of these cells [8]. Therefore, it is thought that the use of targeted MSCs by molecular methods will provide a higher rate of tissue repair for migration to the target organ or tissue.

Recent studies have shown that MSCs overexpressing inflammatory chemokine receptors such as CC chemokine receptor 2 (CCR2) have a higher rate of migration to the inflammatory tissue [9,10]. Inflammatory chemokine receptor expression is an important strategy for direct delivery to target tissue by gaining MSCs with advanced molecular techniques [11,12].

In this study, the therapeutic effect of CCR2-overexpressing gingiva MSCs (GMSCs) was investigated in an experimental animal model of acute colitis. The use of gingival MSCs targeted by molecular methods is intended to provide a higher immunomodulatory effect for migration to the target organ or tissue.

## Materials and methods

### Ulcerative colitis animal model

In this study, 28 male Wistar Albino rats (2–3 months old) were purchased from the Experimental Animal Center of the Muğla Sıtkı Koçman University. All protocols applied on animals was approved by the Ethical Committee of Experimental Animals Center of the Muğla Sıtkı Koçman University with the approval number of 24/21. Rats were housed in animal cages at the Experimental Animals Center of the Muğla Sıtkı Koçman University. Rats were fed according to their daily feed which has 16% protein content and 4–5% fat content (approximately 30–40 g pellet/day) and water requirements. The laboratory environment was kept at 20–25°C with humidity levels between 30% and 50%, and 12 hours of light/dark cycle. An experimental model for ulcerative colitis, which is one of the experimental models that best mimics ulcerative colitis, was performed as described before [13]. In brief; colitis was induced by a single intrarectal administration of 500 μl of 4% acetic acid (v/v) on day 0. GMSCs or CCR2^+^GMSCs were administered to rats intraperitoneally, on day 8. On days 8 and 29, rats’ weight loss and disease activity index were evaluated. Groups were as follows; Group 1 (Healthy Control), Group 2 (Colitis Model), Group 3 (Colitis+GMSCs 2x10^6^), and Group 4 (Colitis+CCR2^+^GMSCs 2x10^6^). Animals were sacrificed by decapitation on day 21 after the administration of MSCs. For the histopathological evaluation and immunological analysis, intestinal tissue samples were collected by cutting the colon with a scalpel immediately after sacrification.

For the experimental study, the sample size was calculated by G-Power analysis as follows; F-test values for calculating the least possible number of animals by choosing 0.75 with an effective value greater than 0.50 as suggested for clinical studies for the analysis of statistical data [14]. The power was calculated as 0.80, type I error as 0.05, and the number of groups as 4, and the total number of animals was determined as 28. The number of animals in each group was 7. The scheme for the experimental model is given in S1a Fig.

### Isolation and characterization of GMSCs

Rat GMSCs from the second passage in the stocks of Muğla Sıtkı Koçman University Research Laboratories Center Immunology and Stem Cell Laboratory were used by culturing them into the third passage. Previously isolated MSCs were obtained from the oral cavity of rats, from the gingiva between the incisor and molar teeth. GMSCs were cultured in DMEM medium containing 1% penicillin/streptomycin (100 IU/mL, 100 µg/mL) in a 5% CO2 incubator at 37°C. Cells reaching 80–90% confluency were trypsinized with 0.25% trypsin EDTA for 4 minutes at 37°C, removed from the bottom, washed twice with 5 mL of DMEM medium containing 10% fetal bovine serum (FBS), centrifuged at 1200 rpm for 5 minutes, the supernatant was discarded, and the cell pellet at the bottom was counted with a hemocytometer. The cells were cultured (6.000 cells/cm2) in the same culture medium in T75 flasks in the third passage.

GMSCs in the third passage were stained with anti-CD29 (PE), anti-CD73 (APC), anti-CD90 (PerCp) antibodies for the analysis of positive surface markers, and anti-CD14 (PE), anti-CD34 (APC), anti-CD45 (FITC) antibodies for the analysis of negative markers via flow cytometry (BD Accuri C6 Plus software).

### CCR2 overexpression in GMSCs

The overexpression of CCR2 on GMSCs was performed as described previously [15]. GMSCs in the third passage were cultured in the presence of lentivirus (plenti-EF1a-mCherry-P2A-Puro-CMV-CCR2) for CCR2 overexpression. In brief, the cells were cultured in a T75 culture plate until 80% confluency, and 60 μg/mL polybrene lentivirus was used for 16 hours of lentiviral transduction, followed by 72 h incubation of gingival MSCs with 1.5 μg/mL puromycin. MSCs were cultured in two groups as follows; 1) CCR2 overexpressing GMSCs (CCR2+GMSCs), 2) non-CCR2 GMSCs (only vector; plenti-EF1a-mCherry-P2A-Puro GMSCs) before the experimental studies.

After the culture period, CCR2^+^ GMSCs were analyzed for the expression of chemokine receptors. For the flow cytometer analysis, GMSCs (100,000 cells) were suspended in 100 µL of phosphate buffer solution (PBS) and analyzed within the FL3 channel for mCherry signal. A negative histogram was determined by analyzing GMSCs cultured without plasmid. CCR2 expression was analyzed by staining cells with anti-CCR2 antibody (FITC). The histogram data was given as a percentage (%) of the cells in the total gated cell population. CCR2 gene expression was analyzed by real-time PCR analysis. After RNA isolation of the cells with the RNA isolation kit, DNase treatment was performed to purify the obtained RNAs from genomic DNA contamination. Then, cDNA synthesis was performed with random hexamer primers by applying the kit protocol. For this, 4 µL template RNA was incubated at 37°C for 1 hour and 95°C for 3 minutes for the reverse transcription reaction. After cDNA was obtained, Real-Time PCR reactions were performed with gene-specific primers (CCR2) and control primer (beta-actin). 5 μl of reverse transcript cDNA template was used for the PCR reaction with an autofluorescent quantitative PCR device. 2X SYBR Green master mix, gene-specific primers, and template DNA were used in the Real-Time PCR reaction mixture. Reaction mixes were prepared with final concentrations of 1X for SYBR green mix, 0.05–0.5 µM for primers, and 0.1–10 ng for cDNA. A 3-step Real-Time PCR program was used. Expression levels were calculated by evaluating the Cp values and melting curves taken after the experiment. Primers used for CCR2 were as follows; forward primer 5′-TGC TCT AGA GAA GAC AAT AAT ATG TTA CC-3′, reverse primer 5′-ATA GCG GCC GCT TAC AAC CCA ACC GAG ACC T-3′.

### Assessment of disease activity index in experimental colitis model

The evaluation of the disease activity index (DAI) was done by scoring body weight loss and feces. DAI was done as described previously [16]. Body weight change values were normalized as a percentage of the body weight at the beginning of the experiment on day 0. Less than 5% body weight loss was scored as 0 points, 1–5% body weight loss was scored as 1 point, 5–10% body weight loss was scored as 2 points, 10–15% body weight loss was scored as 3 points, over 15% body weight loss was scored as 4 points. Feces evaluation was done as follows; feces consistency (0 points, formed pellets; 2 points, pasty/semi-formed feces; and 4 points, liquid feces) and rectal bleeding (0 points, no rectal bleeding; and 4 points, visible major bleeding). Evaluation of DAI is given in **Table 1**.

**Table 1:** Evaluation of the disease activity index.

Score	Decrease in body weight (%)	Feces consistency	Feces appearance/Rectal bleeding
0	0	Normal (formed pellets)	Normal
1	1-5	Normal (formed pellets)	Occult blood +
2	5-10	Loose feces	Occult blood ++
3	10-15	Loose feces	Occult blood +++
4	>15	Diarrhea	Gross bleeding

### Analysis of cytokine secreting T lymphocytes

Inflammatory cell subsets that play a role in IBD are Th1 and Th17 lymphocytes. In order to analyze Th1 and Th17 lymphocytes in the intestinal area, the cytokines they secrete were analyzed by performing intracellular cytokine secretion analysis. After sacrification, half of the intestinal samples of the rats were used for IFN-γ or IL-17 secreting T lymphocytes analysis. The lymphocyte isolation protocol for intestinal samples was performed as described before [17]. In brief; the intestinal tissues were cut into 0.2–0.5 mm pieces with a scalpel, and enzymatically digested with 5 mL of collagenase type I solution (100 U/ml) by incubating at 37°C for 45 minutes. After incubation period, cells were suspended in a DMEM medium containing 10% FBS and 1% Penicillin/Streptomycin and centrifuged at 1500 rpm for 5 minutes. The supernatant was discarded and the cell pellet at the bottom was stimulated with PMA and ionomycin 5 µg/mL at 37°C for 4 hours. At the end of the culture period, cells were labeled with anti-rat-CD4, anti-rat-IFNγ, anti-rat-IL17 antibodies and analyzed via flow cytometry.

### Histopathological evaluation

In order to evaluate the healing parameters in the intestine, ½ of the intestinal tissue samples were transferred into 10% formaldehyde. The tissues were embedded in paraffin and 4–5 micron sections were taken, and the sections were stained with hematoxylin-eosin after deparaffinization. The tissue sections were examined for epithelial cell morphology, tissue integrity, ulcers, inflammation, and fibrosis. Fibrosis was scored as follows: no; 0, mild; 1, moderate or severe; 2. Mucosal integrity was scored as follows: preserved mucosa; 0, mucosal loss; 1. İnflammation was scored as follows; 0: No inflammation or mild chronic inflammation, 1: Moderate chronic inflammation, 2: mild acute inflammation, 3: moderate to severe acute inflammation. Ulcers were scored as follows; no ulcers: 0, spot ulcer: 1, moderate ulcers: 2, severe ulcers: 3. Finally, grading was done according to Nancy index [18]. Ulcer; Grade 4, acute inflammation moderate or severe: Grade 3, mild; Grade 2. Chronic inflammation (no acute inflammation) moderate or severe: Grade 1, mild or no; Grade 0. Quantitative results were expressed according to inflammatory cells (neutrophils, lymphocytes). The scoring system is given in S2 Fig.

### Homing analysis of MSCs

To determine the homing ratio of CCR2^+^GMSCs or GMSCs in the intestinal tissue, tissue sections were analyzed by fluorescent microscope. 630 nm emission filter was used to image the mCherry labeled cells in tissue sections. Images were analyzed by obtaining fluorescence intensity.

### Statistical analysis

The statistical analysis was done by using Graphpad Prism 8.0 version. Data were given as mean (mean) ± standard deviation (SD) (minimum-maximum) values in each group. Comparison of data from more than two groups was done with ANOVA test (one-way analysis of variance). Un-paired Student’s t-test was used for comparison between the two groups. Pathological scores were analyzed with Mann-Whitney U tests or Kruskal-Wallis test for multiple independent samples of non-parametric tests. p < 0.05 was considered statistically significant.

## Results

### Characterization of GMSCs

GMSCs expressed CD29, CD73, and CD90 positive markers over 95% and lack the expression of CD14, CD34, and CD45 negative markers. The data for flow cytometry analysis is given in S1b Fig.

### Evaluation of Allo-Rejection after administration of GMSCs

The peripheral organs of the rats were macroscopically observed for complications of MSCs administration during the study period and after sacrification. No complications for MSCs application such as tumorigenesis in the peripheral organs or allo-rejection were observed after the administration of GMSCs or CCR2+GMSCs in the rats during the experimental study period.

Animals were observed for survival throughout the study period. It was recorded that no animals died until the day of sacrifice.

### GMSCs showed high expression of CCR2 after transduction

**Fig 1:**
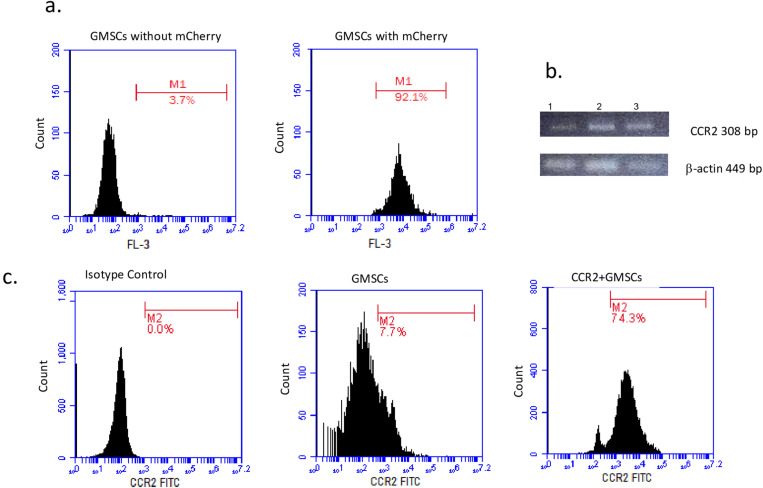
Chemokine receptor expression analysis. The representative flow cytometry analysis is as follows; a) The third passage GMSCs without mCherry carrying plasmid is shown by flow cytometry histogram analysis in FL-3 channel, b) The third passage GMSCs were analyzed after transduction with mCherry carrying CCR2 plasmid. c) Flow cytometry analysis of GMSCs without CCR2 and after transduction with CCR2 plasmid is shown. The histogram analysis is done with isotype control IgG antibody. The histogram analysis showed high expression of CCR2 on GMSCs after the transduction process. All of the analyses were done in three replicates.

MSCs were transduced with a lentiviral vector encoding CCR2-mCherry to overexpress CCR2 or only vector with mCherry to use as a control. When the mCherry signal of GMSCs was analyzed in the FL-3 channel by flow cytometry, it was observed that the fluorescence intensity of GMSCs carrying mCherry was higher (92.1%) compared to GMSCs not carrying mCherry (3.7%) (**Fig 1a**). Also, we examined the mRNA levels for CCR2 expression in GMSCs after transduction with lentiviral vectors. It was observed that mRNA level was relatively high in GMSCs transduced with CCR2 lentiviral vector (3.2 folds), compared to control GMSCs culture (1.0 fold) (**Fig 1b**). 16 hours after the transduction process, we observed upregulated expression of CCR2 (74.3%) in GMSCs transduced with CCR2 lentiviral vector, compared to control GMSCs culture (7.7%) in the flow cytometry analysis (**Fig 1c**).

### The disease activity index decreased with the administration of CCR2^+^GMCSs

**Fig 2:**
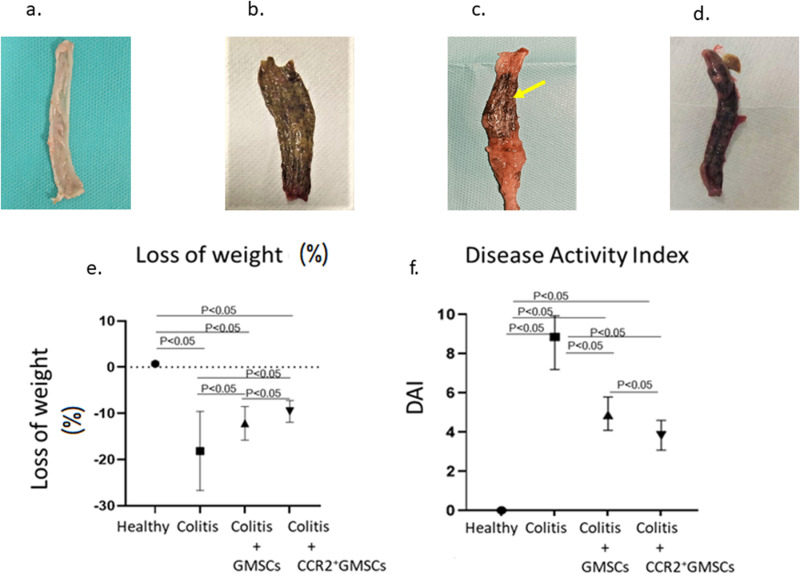
Macroscopic evaluation of colons. **a)** Colon image of healthy group. No mucosal lesion is observed in the colon. b) Colon image of experimental colitis model (Group 2) on day 29. Mucosal lesions appear in the entire colon. c) Colon image of GMSCs administered experimental colitis model (Group 3) on day 29. Mucosal lesions in the colon appear to be partially located in the colon (yellow arrow). d) Colon image of CCR2^+^GMSCs administered experimental colitis model (Group 4) on day 29. In the macroscopic evaluation, no mucosal lesion is observed in the colon. e) Statistical analysis of disease activity index and percent of weight loss. The weight loss was significantly decreased in Group 3 and Group 4 compared to Group 2 (p < 0.05 and p < 0.05, respectively). The weight loss was remarkably decreased in Group 4, when compared with Group 3, on day 29. e) Disease activity index (DAI) was significantly decreased in Group 3 and Group 4 compared to Group 2 (p < 0.05 and p < 0.05, respectively). DAI was significantly decreased in Group 4, when compared with Group 3, on day 29. There are 7 rats in each group.

All the animals that had received intracolonic acetic acid (4%) developed gross bleeding, loose feces, and diarrhea, which are the symptoms of ulcerative colitis, within 72 hours. In the examination of the colon, ulcerated and hemorrhagic mucosae were observed in all the experimental colitis animals in Group 2, on day 29. Animals in Group 3 showed slight ulcerated and hemorrhagic mucosae, but no ulcers or hemorrhagic mucosae were observed in Group 4. No colonic damage was detected in the healthy control group (**Fig 2a–c**).

The weight loss was significantly decreased in Group 3 (Colitis+GMSCs 2x10^6^) (14.3 ± 4.6 grams) and Group 4 (Colitis+CCR2^+^GMSCs 2x10^6^) (9.8 ± 2.1 grams) compared to Group 2 (Colitis Model) (19.2 ± 9.1 grams) (p < 0.05 and p < 0.05, respectively). The weight loss was remarkably decreased in Group 4, when compared with Group 3, on day 29. No bleeding, but loose feces in two animals was observed in Group 4, on day 29. Disease activity index (DAI) was significantly decreased in Group 3 (5.1 ± 0.8) and Group 4 (3.9 ± 0.6), compared to Group 2 (8.7 ± 1.2) (p < 0.05 and p < 0.05, respectively). DAI was significantly decreased in Group 4, when compared with Group 3, on day 29 (p < 0.05) (**Fig 2d**,**2e**) (**Table 2**).

**Table 2:** DAI scores for each animal.

Healthy	Colitis	Colitis+GMSCs	Colitis+CCR2 + GMSCs
0	8	4	3
0	6	6	4
0	11	4	4
0	9	4	3
0	8	5	3
0	13	5	5
0	9	4	4

### Intestinal IFN-γ and IL-17 secreting CD4+ T cells downregulated with GMSCs and CCR2+GMSCs

**Fig 3:**
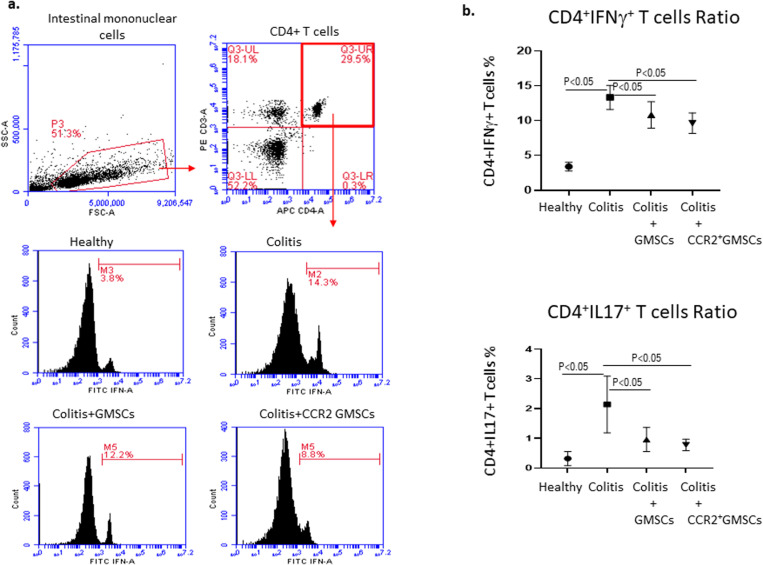
Cytokine secreting CD4+ T lymphocyte ratios. **a)** Representative images for gating strategy in flow cytometry analysis is given. The flow cytometry analysis is done for Th1 (IFN- **γ**+CD4+ cells), and Th17 (IL17+CD4+ cells). Lymphocytes were gated (P3) from total mononuclear cells isolated from colons. P3 gate was analyzed for CD3+CD4+ T cells (T helper cells) by dot plot analysis (upper right quadrant). CD3+CD4+ T cells were analyzed for IFN-γ or IL-17 ratios by histogram analysis. b) Statistical analysis of IFN-γ or IL-17 secreting cells. CD4+IFN-γ+ T cells and CD4+IL17+ T cells were significantly high in Group 2 compared to Group 1 (p < 0.05 and p < 0.05, respectively). The intraperitoneal injection of GMSCs and CCR2+GMSCs significantly reduced CD4+IFN-γ+ and CD4+IL17+ T cell ratios compared to Group 2 (p < 0.05 and p < 0.05, respectively). Comparison of data from more than two groups was done with ANOVA test (one-way analysis of variance). Unpaired Student’s t-test was used for comparison between the two groups. Flow cytometry analyzes were performed as single analyses.

Th1 and Th17 mediated immunity constitute the dominant immune responses in the pathogenesis of ulcerative colitis. Therefore, we evaluated the ratios of intestinal IFN-**γ** secreting CD4+ T cells to demonstrate Th1 lymphocyte subsets, and IL-17 secreting CD4+ T cells to demonstrate Th17 lymphocyte subsets. The ratio of IFN-**γ** secreting CD4+ T cells was significantly decreased in Group 3 (11.3 ± 1.4%) and Group 4 (9.2 ± 1.1%), compared to Group 2 (13.7 ± 1.2%) (p < 0.05 and p < 0.05, respectively). IL-17 secreting CD4+ T cells ratio was notably decreased in Group 3 (0.9 ± 0.6%) and Group 4 (0.8 ± 0.2%), compared to Group 2 (2.2 ± 0.8%) (p < 0.05 and p < 0.05, respectively). Group 2, Group 3, and Group 4 showed significantly high ratios of IFN-γ or IL-17 secreting CD4 + T lymphocytes, when compared with the healthy control group (IFN-γ+CD4+T cells: 3.9 ± 0.6%, IL-17+CD4+T cells: 0.3 ± 0.2%) (p < 0.05, p < 0.05, and p < 0.05, respectively) (**Fig 3**).

### CCR2+GMSCs demonstrated colonic epithelial integrity by highly migrating to colon tissue

**Fig 4:**
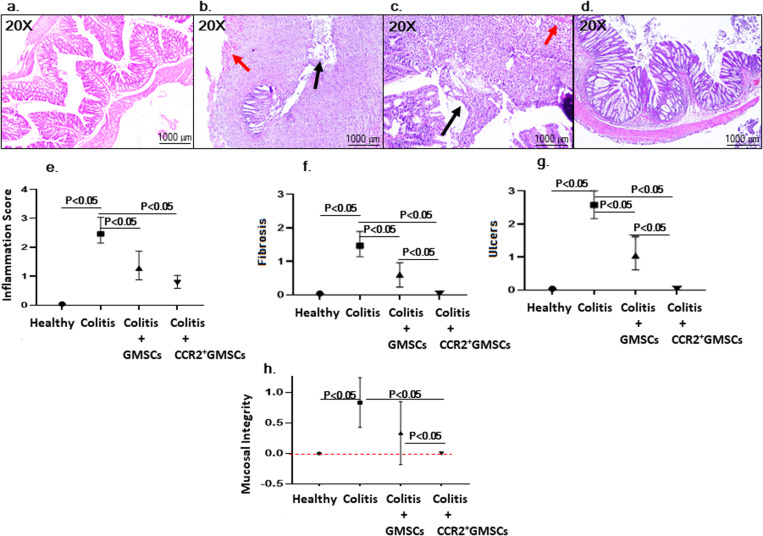
Histopathological analysis of colon tissues. Light microscopy images from hematoxylin-eosin staining of tissue slices obtained from Group 1 (Healthy Control) rat colon (a) which was scored as Grade 0 according to Nancy index, whereas Colitis rat (Group 2) showed Fibrosis (red arrow) and ulcers (black arrow) which was scored as Grade 4 (b), the Colitis rat treated with GMSCs (Group 3) showed fibrosis and ulcers which was scored as Grade 4 (c), and Colitis rat treated with CCR2 + GMSCs (Group 4) showed no fibrosis, inflammation, or ulcers in the colon which was scored as Grade 0 (d). e) Statistical analysis for inflammation scoring shows that inflammation was significantly reduced in Group 3 and Group 4 compared to Group 2 (p < 0.05). f) Statistical analysis for fibrosis demonstrates that CCR2+GMSCs (Group 4) significantly reduced fibrosis compared to GMSCs (Group 3) and no treatment subjects (Group 2). g) Statistical analysis for ulcers demonstrates that CCR2+GMSCs (Group 4) significantly reduced ulcers compared to GMSCs (Group 3) and no treatment subjects (Group 2) (p < 0.05). h) Statistical analysis for mucosal integrity shows that CCR2+GMSCs (Group 4) significantly improved mucosal integrity compared to Group 2 and Group 3 (p < 0.05). There are 7 rats in each group. Pathological scores were analyzed with Mann-Whitney U test for multiple independent samples of non-parametric tests.

Colonic epithelial cells play a crucial role by maintaining the integrity of the intestinal barrier, mucus production, and the release of antimicrobial peptides [19]. We, therefore, investigated the effects of CCR2+GMSCs or GMSCs in colon samples by histopathological evaluation. Inflammation scores significantly reduced in Group 3 (1.2 ± 0.6) and Group 4 (0.8 ± 0.2), compared to Group 2 (2.6 ± 0.4) (p < 0.05 and p < 0.05, respectively). Fibrosis and ulcers significantly reduced in Group 3 (Fibrosis: 0.6 ± 0.4, ulcers: 1.0 ± 0.6) and Group 4 (Fibrosis: 0.0 ± 0.0, ulcers: 0.0 ± 0.0) compared to Group 2 (Fibrosis: 1.6 ± 0.4, ulcers: 2.6 ± 0.4) (Fibrosis p < 0.05 and p < 0.05, ulcers p < 0.05 and p < 0.05, respectively). Fibrosis and ulcer scores in Group 4 were statistically close to Group 1 (Fibrosis: 0.0 ± 0.0, ulcers: 0.0 ± 0.0), and no significant difference was observed between Group 4 and Group 1 (p < 0.05). In addition, fibrosis and ulcers significantly reduced in Group 4 compared to Group 3 (p < 0.05). Mucosal integrity significantly improved in Group 4 (0.0 ± 0.0) compared to Group 2 (0.8 ± 0.2) (p < 0.05) and Group 3 (0.2 ± 0.8) (p < 0.05). Grade of UC reduced in 6 out of 7 animals in Group 4 (Grade 2), but 4 animals in Group 3 and 7 animals in Group 2 were Grade 4 according to Nancy Index (**Fig 4**).

**Fig 5:**
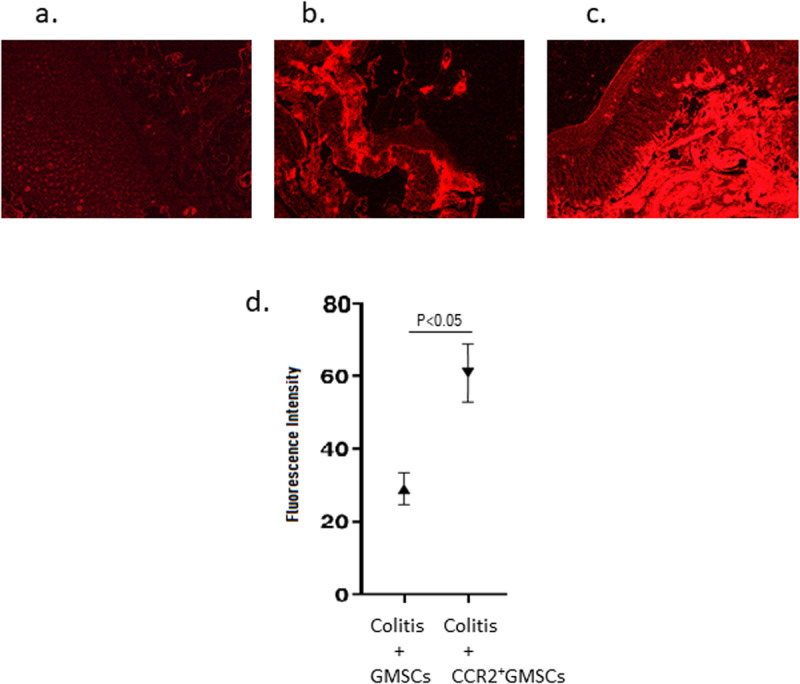
Homing analysis of GMSCs. **a)** GMSCs without mCherry label were injected into an experimental colitis rat to determine the fluorescence intensity. No fluorescence signal was detected in the microscope analysis at 630nm emission, b) mCherry labeled GMSCs migrated to the colon with fluorescence intensity of 19.6 ± 9.8, c) mCherry labeled CCR2^+^GMSCs migrated to with fluorescence intensity of 61.2 ± 13.7. d) Statistical analysis of fluorescence microscopy results for GMSCs and CCR2^+^GMSCs. The fluorescence intensity values of the treatment group with CCR2^+^GMSCs (Group 4) were significantly higher in inflamed colon tissues compared to the treatment group with GMSCs (Group 3) (p < 0.05). There are 7 rats in each group. Microscopy results were analyzed with Mann-Whitney U tests for multiple independent samples of non-parametric tests.

We analyzed homing rate of mCherry labeled GMSCs into intestinal tissue by analyzing fluorescence intensity under fluorescent microscopy. The fluorescence intensity in the intestine was significantly high in Group 4 (61.2 ± 13.7) compared to Group 3 (19.6 ± 9.8) (p < 0.05), and no fluorescence signal was detected in Group 2 (0.0 ± 0.0) (p < 0.05) (**Fig 5**).

## Discussion

Ulcerative colitis (UC) is a major form of inflammatory bowel disease (IBD) characterized by chronic inflammation and ulcers of the colonic mucosa with a variable spread from the rectum to the cecum [19]. Although the first-line treatment is 5-aminosalicylic acid, corticosteroids, immunosuppressive drugs and various biological agents such as anti-tumor necrosis factor-alpha (TNF-α) antibodies, anti-integrin antibodies, anti-interleukin (IL) 12–23 antibodies are used in patients who do not respond to this treatment [20,21]. However, some patients with UC still remain symptomatic despite optimal doses of oral 5-aminosalicylic acid drugs, steroids, and systemic corticosteroids [22]. Therefore, there is still a need for new treatment options that can target inflammatory responses while simultaneously promoting damaged tissue repair.

CCR2 plays an important role in the extravasation and migration of monocytes into tissue during inflammation. A recent study showed that CCR2 promotes the migration of monocytes into inflammatory bowel tissue in a mouse model of colitis [23]. Also, Chemokine (C-C motif) ligand 2 (CCL2)-CCR2 axis is important in involving immune cells in tumor progression [24]. Therefore, overexpression of CCR2 may be an effective strategy for cells to migrate to the target tissue at a higher amount to show high regenerative effects.

In the present study, we investigated the regenerative and anti-inflammatory effects of CCR2 overexpressing GMSCs on the UC animal model. The results showed that GMSCs and CCR2^+^GMSCs equally downregulated Th1 and Th17 responses in the inflamed intestinal tissues of UC model rats. However, CCR2^+^GMSCs demonstrated a high regenerative capability in the UC animal model compared to non-genetically modified GMSCs. Lesions in UC mostly have an inflammatory microenvironment dominated by cytokines such as interleukin (IL) 1β, IL-6, IL-17A, and IL-21 [25]. MSCs exert anti-inflammatory responses by interacting with the immune cells in tissues as well as with paracrine factors secreted in the tissues during immune responses [26]. One of the paracrine factors in inflamed tissue is chemokines. MSC migration is induced by the interaction of chemokine ligands with their corresponding receptors [27]. Therefore, overexpression of chemokine receptors on cells for optimal delivery of MSCs, particularly by intravenous or intramuscular injections, is an effective strategy for a higher migration rate of MSCs into inflamed tissue [28]. In this study, we observed that both CCR2^+^GMSCs and GMSCs notably decreased IFN-**γ** or IL-17 secreting CD4+T lymphocytes when compared with untreated colitis rats. CCR2^+^GMSCs tended to decrease CD4+IFN-γ+ T lymphocyte ratios in comparison with GMSCs without chemokine receptors in inflammatory bowel tissue, but there was no statistically significant difference. Previous studies on UC have shown that MSCs can promote colonic immunoregulation by secreting tumor necrosis factor-α-induced gene/protein 6 (TSG-6), inducing macrophage phenotypic transition from M1 to M2, and also has been shown to modulate T and B lymphocytes by secreting transforming growth factor-beta (TGF-β), indoleamine dioxygenase (IDO) and prostaglandin E2 (PGE2) as anti-inflammatory mediators [4]. A recent study demonstrated that a single injection of human umbilical cord MSCs (hUCMSCs) reduced the colon inflammation score when compared with multiple injections of bone marrow MSCs (BMSCs) [26]. The results of our study on inflammatory responses in colitis showed that GMSCs have anti-inflammatory effects with or without chemokine receptors. In the histopathological evaluation, it was observed that CCR2^+^GMSC and GMSCs decreased inflammatory cell scores in the colon tissue, which supported the results of inflammatory cell responses in immunological analyses. These results are compatible with the previous studies. However, the histopathological analysis demonstrated that CCR2+GMSCs significantly reduced fibrosis and ulcers in colon tissues compared to GMSCs. To further investigate how CCR2^+^GMSCs induce higher colonic regeneration, we analyzed the distribution of GMSCs in inflammatory colon tissues. The results showed that homing amounts were higher in CCR2^+^GMSCs when compared with GMSCs. Previous studies demonstrated that pre-treatment of MSCs with CXCL12 enhances their migration towards inflamed or injured tissue and increases the secretion of growth factors such as vascular endothelial growth factor or fibroblastic growth factor [28–30]. In similar, CXCR-4 gene overexpressed BMSCs (CXCR-BMSCs) exerted increased homing of BMSCs into the colon in the colitis murine model [31]. In a previous study on ischemic stroke in rats, CCR2 overexpressed BMSCs enhanced migration to the ischemic hemisphere and improves the therapeutic outcomes [32]. Our results are compatible with previous studies that CCR2+GMSCs have high regenerative capacity in UC by migrating in large quantities to the inflamed colon.

Compared with previous studies, similar to the results obtained in this study, MSCs may be a therapeutic option for IBD by regulating intestinal immune responses, reducing fibrosis, and may also have a regenerative effect by inducing angiogenesis. In addition, MSC-derived secretomes, such as exosomes or paracrine factors, have therapeutic potential in IBD as they exhibit effects similar to MSCs [33]. In addition to the studies on the therapeutic effects of MSCs alone in IBD, current studies are aimed at increasing the therapeutic effect of MSCs by preconditioning or genetically engineering the MSCs. A recent study demonstrated that preconditioning hUCMSCs with modified neuronal medium resulted in enhanced therapeutic effects of MSCs on colitis through PGE2-Mediated T-Cell modulation of intestinal tissue in IBD [34]. As well as preconditioning the MSCs, targeting these cells to inflammatory tissue is another strategy for the delivery of MSCs in high amounts to the injured sites. Previous studies on targeting MSCs to injury sites have shown that nongenetically engineering methodologies to enhance stem cell delivery, such as through the enzymatic modification of cell surface glycoproteins or by biotinylation of cell surface proteins may be a novel cell targeting method [35,36]. Not the same methodology but a similar effect is observed in our study that CCR2 overexpression of GMSCs resulted in the migration of these cells in high amounts to injured intestinal tissue in the UC animal model. Although, studies reveal a safe use of lentiviral vectors [37], a limitation of this study is lentiviral vectors may not be used in humans. Therefore, further studies are needed for the human application of lentiviral-infected MSCs for the treatment of colitis or other related diseases.

In conclusion, the overexpression of CCR2 on GMSCs has modulatory effects on immune responses in UC and has high regenerative capability by enhancing migration towards inflammatory tissue sites in the colon. Increasing chemokine receptor expression of GMSCs may be an alternative to cellular therapies in UC.

## Supporting information

S1 Fig a) The study design. Wistar albino rats received 4% (w/w) acetic acid intrarectally on day 0. GMSCs or CCR2+GMSCs were administered intraperitoneally on day 8. Rats were evaluated for disease activity index on days 8 and 29. Rats were sacrificed on day 29 (21 days after treatment with GMSCs). Totally 28 rats were included in the study. b) Characterization of GMSCs. In the third passage, GMSCs were analyzed for positive and negative markers. GMSCs showed a high expression ratio for positive markers (CD29, CD73, and CD90). GMSCs lack the expression of negative markers for MSCs (CD14, CD34, and CD45). All of the analyses were done in three replicates.
(TIF)

S2 Fig Nancy Index. The scoring system is given as described previously [38].
(TIF)

S1 Data Raw data for colitis evaluation. (DOCX)
