# Supplementary figures and images for: CCR2 overexpressing gingiva mesenchymal stem cells provide high intestinal regeneration in a rat model of ulcerative colitis
Source: PLoS One. 2025 Jun 5;20(6):e0325566. doi: 10.1371/journal.pone.0325566 (PMC12140208; doi:10.1371/journal.pone.0325566)

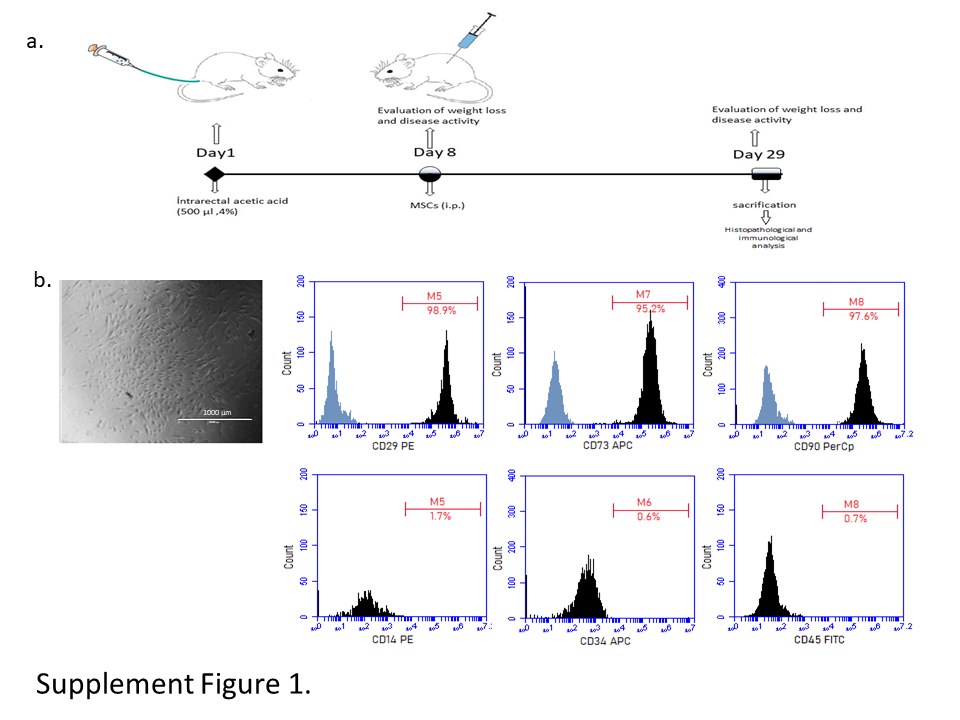

Supplement: S1 Fig — Wistar albino rats received 4% (w/w) acetic acid intrarectally on day 0. GMSCs or CCR2+GMSCs were administered intraperitoneally on day 8. Rats were evaluated for disease activity index on days 8 and 29. Rats were sacrificed on day 29 (21 days after treatment with GMSCs). Totally 28 rats were included in the study. b) Characterization of GMSCs. In the third passage, GMSCs were analyzed for positive and negative markers. GMSCs showed a high expression ratio for positive markers (CD29, CD73, and CD90). GMSCs lack the expression of negative markers for MSCs (CD14, CD34, and CD45). All of the analyses were done in three replicates. (TIF) [file pone.0325566.s001.TIF]

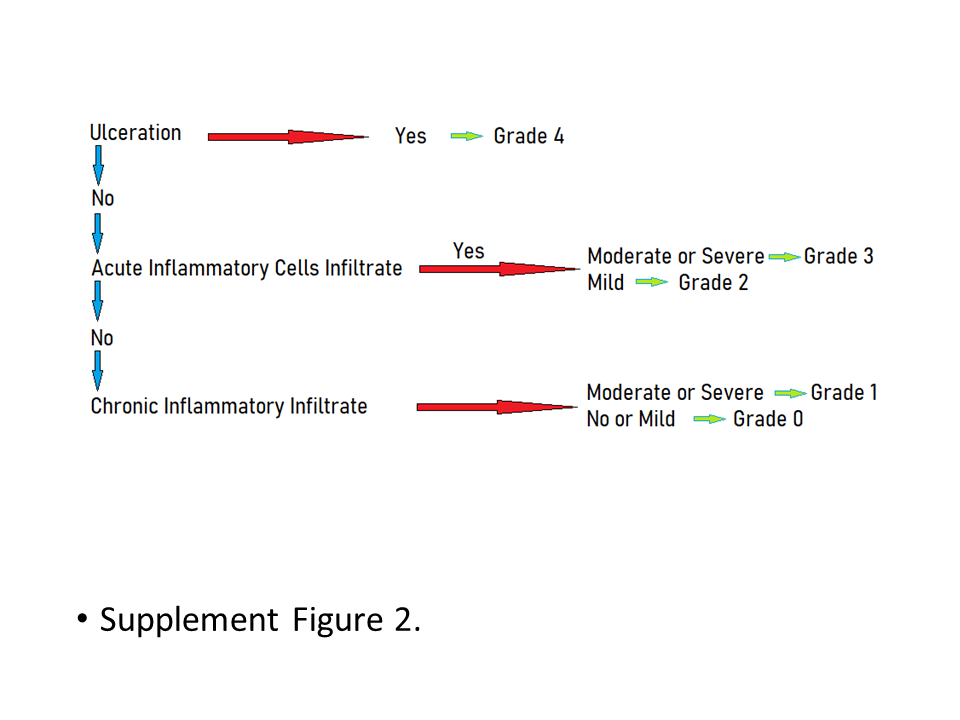

Supplement: S2 Fig — The scoring system is given as described previously [38]. (TIF) [file pone.0325566.s002.TIF]
