# Supplementary material for: CCR2 overexpressing gingiva mesenchymal stem cells provide high intestinal regeneration in a rat model of ulcerative colitis
Source: PLoS One. 2025 Jun 5;20(6):e0325566. doi: 10.1371/journal.pone.0325566 (PMC12140208; doi:10.1371/journal.pone.0325566)
Supplement: S1 Data — (DOCX) [file pone.0325566.s003.docx]

Raw Data for Colitis Evaluation

1. Mucosal Integrity

| Healthy | Colitis | Colitis+GMSCs | Colitis+CCR2+GMSCs |
| --- | --- | --- | --- |
| 0 | 1 | 1 | 0 |
| 0 | 1 | 1 | 0 |
| 0 | 1 | 0 | 0 |
| 0 | 1 | 0 | 0 |
| 0 | 0 | 0 | 0 |
| 0 | 1 | 0 | 0 |

1. Disease Activity Index

| Healthy | Colitis | Colitis+GMSCs | Colitis+CCR2+GMSCs |
| --- | --- | --- | --- |
| 0 | 8 | 4 | 3 |
| 0 | 6 | 6 | 4 |
| 0 | 11 | 4 | 4 |
| 0 | 9 | 4 | 4 |
| 0 | 8 | 5 | 3 |
| 0 | 13 | 5 | 5 |
| 0 | 7 | 4 | 4 |
